# How do combinations of unhealthy behaviors relate to attitudinal factors and subjective health among the adult population in the Netherlands?

**DOI:** 10.1186/s12889-020-8429-y

**Published:** 2020-04-03

**Authors:** Charlotte M. Dieteren, Werner B. F. Brouwer, Job van Exel

**Affiliations:** 1 Erasmus University Rotterdam, Erasmus School of Health Policy & Management, P.O. Box 1738, 3000 DR Rotterdam, the Netherlands; 2 Erasmus University Rotterdam, Erasmus School of Economics, Rotterdam, The Netherlands

**Keywords:** Health behaviours, Clustering risk attitude, Time orientation, Subjective health

## Abstract

**Background:**

Health behaviours like smoking, nutrition, alcohol consumption and physical activity (SNAP) are often studied separately, while combinations can be particularly harmful. This study aims to contribute to a better understanding of lifestyle choices by studying the prevalence of (combinations of) unhealthy SNAP behaviours in relation to attitudinal factors (time orientation, risk attitude) and subjective health (self-rated health, life expectancy) among the adult Dutch population.

**Methods:**

In total 1006 respondents, representative of the Dutch adult population (18–75 years) in terms of sex, age, and education, were drawn from a panel in 2016. They completed an online questionnaire. Groups comparisons and logistic regression analyses (crude and adjusted) were applied to analyse (combinations of) SNAP behaviours in relation to time orientation (using the Consideration of Future Consequences scale comprising Immediate (CFC-I) and Future (CFC-F) scales) and risk attitude (Health-Risk Attitude Scale; HRAS-6), as well as subjective health (visual analogue scale and subjective life expectancy).

**Results:**

In the analyses, 989 respondents (51% men, average 52 years, 22% low, 48% middle, and 30% high educated) were included. About 8% of respondents engaged in four unhealthy SNAP behaviours and 18% in none. Self-rated health varied from 5.5 to 7.6 in these groups, whilst subjective life expectancy ranged between 73.7 and 85.5 years. Logistic regression analyses, adjusted for socio-demographic variables, showed that smoking, excessive drinking and combining two or more unhealthy SNAP behaviours were significantly associated with CFC-I scores, which increased the odds by 30%, 18% and 19%, respectively. Only physical inactivity was significantly associated with CFC-F scores, which increased the odds by 20%. Three out of the four SNAP behaviours were significantly associated with HRAS-6, which increased the odds between 6% and 12%. An unhealthy diet, excessive drinking, and physical inactivity were significantly associated with SRH, which decreased the odds by 11%. Only smoking was significantly associated with subjective life expectancy, which decreased the odds by 3%.

**Conclusion:**

Our findings suggest that attitudinal factors and subjective health are relevant in the context of understanding unhealthy SNAP behaviours and their clustering. This emphasizes the relevance of a holistic approach to health prevention rather than focusing on a single unhealthy SNAP behaviour.

## Background

The effect of lifestyle on morbidity and mortality is increasingly being recognized [1–3]. The disease burden attributed to lifestyle choices primarily consists of non-communicable diseases (NCDs). May et al., [4] among others, have shown that making healthy choices regarding smoking, nutrition, alcohol consumption and physical activity (SNAP) (here used to define lifestyle), has a strong impact on the prevention of NCDs. However, contrary to what would be desirable from a public health perspective, studies have shown that adherence to a healthy lifestyle (making healthy choices) has decreased over the past decade [5, 6].

Adherence to a healthy lifestyle may have decreased in general, however in the last decade a strong reduction in the prevalence of smoking has been observed. Over 20% of the worldwide population smokes, which leads to high numbers of premature deaths [7]. Nutrition also plays a major role in premature deaths and disability. It has been estimated that in 2017 a poor diet was a risk factor in one in five of all deaths globally [8]. Excessive alcohol intake has been linked to 3 million deaths in 2016 [9]. Furthermore, almost a quarter of the adult population is physically inactive. Sedentary lifestyles are increasing in varying rates across countries, but seem to currently be most persistent and alarming in developed countries [10].

Healthy lifestyle promotion requires a comprehensive understanding of the way people behave. Mostly, unhealthy lifestyle choices do not occur in isolation, but in different combinations [11]. Engaging in a combination of unhealthy behaviours has been shown to have an additional negative influence on health [12, 13]. A holistic approach to lifestyle interventions may therefore result in more health gains.

Frequent combinations of unhealthy behaviours can be referred to as clusters. Noble et al., [14] conducted a systematic review of the clustering of SNAP health risk factors (referred to from now on as unhealthy SNAP behaviours). They found that the most frequently reported cluster of unhealthy SNAP behaviours was the absence of any of the behaviours, followed by a cluster of excessive alcohol consumption and smoking, a cluster including all behaviours and a cluster with an unhealthy diet and physical inactivity. To understand behavioural choices, it is relevant to have insight into the way unhealthy SNAP behaviours cluster. However, not much research has been conducted on the potential drivers of these clusters.

Our understanding of attitudinal characteristics that influence people’s lifestyle choices remains limited, both in terms of underlying causes and in the way resulting consequences are perceived. Such information can be useful in the context of promoting healthy lifestyles and changing health behaviours. Here, we focus on two attitudinal concepts that may be associated with (the onset of) unhealthy behaviour: time orientation and risk attitude. Various studies show that smokers are less concerned with future consequences of their health behaviour than non-smokers [15–17]. Furthermore, research shows that risk attitude is associated with risky behavioural choices, like smoking [18]. However, associations between these concepts and the engagement in multiple unhealthy SNAP behaviours have not yet been studied. People engaged in multiple unhealthy SNAP behaviours, or in certain combinations of these behaviours, might differ in their attitudinal characteristics.

Engagement in unhealthy SNAP behaviours may also result in (or result from) differences in subjective health experiences and expectations. Subjective health has been shown to be an independent predictor of morbidity and mortality [19, 20] and as such can be considered to carry relevant information in relation to health behaviours. Several studies have shown the association between self-rated health (SRH) and single lifestyle factors [21–23], however few studies have investigated the association between a number (or certain combinations) of healthy lifestyles and SRH [24]. Subjective life expectancy (SLE) is also an indicator for subjective health; it captures how old people expect to become. SLE was found to be associated with smoking behaviour, which may reflect people’s expectations of the increased risk of dying due to smoking, either directly or indirectly through poorer experienced health due to smoking [25]. Associations between SLE and unhealthy dietary choices have also been found [26]. Note that the causal direction between subjective health and unhealthy behaviour can go in both directions. People with an *ex ante* low SLE may for instance be more prone to smoke, as they may expect to have less to loose from smoking. Studying these associations between subjective health and lifestyle factors, while also including behavioural characteristics, and acknowledging that unhealthy behaviours do not occur in isolation has, to our knowledge, not been done before.

Here, we present the results from a study that measured attitudinal factors, subjective health and unhealthy SNAP behaviours simultaneously in the same population. Such information can help to understand potential drivers of unhealthy lifestyle choices, both in terms of causes and consequences of unhealthy behaviours. The objectives of this study were therefore (i) to identify how unhealthy behaviours cluster in a sample representative of the adult population of the Netherlands in terms of sex, age, and education, and (ii) to associate combinations of unhealthy behaviours with attitudinal factors (time orientation, risk attitude) and subjective health (SRH, SLE).

## Methods

### Survey design and sample

In February 2016, cross sectional data were collected through an online survey. The sample was drawn from an online panel representative of the adult population of the Netherlands in terms of age, gender and level of education, between the ages of 18 and 75 years. The survey was distributed until the study population reached an adequate representativeness of the Netherlands (quota sampling). At the beginning of the survey, respondents received information about the purpose of the study and were instructed that participation was voluntary, anonymous to the researchers, and that they could end their participation at any time. When signing up for the panel, members of the panel agreed that by submitting their data at the end of the survey, they were giving permission for the use of their data for the purpose of that study.

### Measures

#### Lifestyle

Lifestyle was operationalized using unhealthy SNAP behaviours, in line with a related study in the Netherlands [27]. Smoking status was assessed and non-smokers were distinguished from occasional smokers (not daily) and current smokers (daily). Respondents were asked to report how many days per week they ate balanced meals: the right proportion, not too much fat, sufficient fruit and vegetables. Respondents were classified as following a healthy diet when they reported eating balanced meals a minimum of 6 days per week [27]. Respondents who reported eating balanced meals less than 6 days per week were classified as following an unhealthy diet. Respondents were asked to report their weekly alcohol consumption. Excessive drinking was defined as consuming six alcoholic drinks or more at least once a week, or when the weekly alcohol consumption exceeded 21 drinks (males) or 14 drinks (females) [28, 29]. Physical activity was measured by asking how often the respondent performed at least 30 min of physical activity (e.g. walking or cycling) per week. People were considered inactive if they performed 30 min of activity on less than 5 days a week [30, 31]. A lifestyle index was computed by adding the number of unhealthy SNAP behaviour present (i.e., smoking, unhealthy diet, excessive alcohol consumption, physically inactive), ranging from 0 (i.e., no unhealthy SNAP behaviour present) to 4 (i.e., all unhealthy SNAP behaviours present). This index has been used before [27].

#### Attitudinal factors

Time orientation was assessed using the consideration of future consequences scale (CFC). The CFC measures the degree to which individuals consider the potentially distant outcomes of current behaviour and whether individuals are influenced by these consequences [32]. The CFC consists of 14 statements, where each statement captures either immediate or future consequences of general behaviour [32, 33]. Respondents were asked to rank the statements on a 5-point Likert scale ranging from “very uncharacteristic for me” to “very characteristic for me”. The CFC score was computed by aggregating item scores (theoretical range 14–70). Research suggests a two factor structure underlying the CFC scale [34–36]. These two factors can be labelled the CFC-Immediate (CFC-I) and CFC-Future (CFC-F) sub-scales. In this study the two factor structure was analysed and reported. Risk attitude in the health domain was measured by a short 6-item version of the Health-Risk Assessment Scale (HRAS-13) [37, 38], the HRAS-6. The HRAS-6 aims to predict how a person will resolve risky health decisions. Respondents were asked to rank six statements on a 7-point Likert scale ranging from “totally disagree” to “totally agree”. The HRAS-6 score was computed by aggregating item scores (theoretical range 6–42), with higher scores indicating stronger risk aversion. The statements of the CFC and the HRAS-6 were presented to respondents in a randomized order.

#### Subjective health

Subjective health was operationalized by eliciting SRH on a visual analogue scale (VAS) (0–10). A score of 10 refers to the best health state imaginable, while a score of 0 refers to the worst health imaginable. As in [27], SLE was obtained through the question: “*What age do you expect to reach?*” The continuous response scale had no minimum score but was limited to 120 years.

### Statistical analysis

SNAP behaviours were used in the analyses in three different ways: as individual health behaviours, as clusters with all potential combinations, and as the lifestyle index. Comparisons between socio-demographics, attitudinal factors, subjective health, and the SNAP behaviours were conducted using Chi-square-tests for categorical variables and one-way analysis-of-variances (ANOVA) for continuous variables. Because multiple tests for significance were performed, an adjusted *p*-value for acceptance and rejection of the null hypothesis was used [39]. A Bonferroni correction was applied, which led to a p-value of 0.001.

We continued with the single SNAP behaviours, striking combinations and the lifestyle index as the main focus. Logistic regression analyses were performed to provide statistical associations. The lifestyle index was dichotomized (with 0 or 1 unhealthy SNAP behaviour present coded as 0; and 2, 3 or 4 unhealthy SNAP behaviours present coded as 1). The lifestyle index was dichotomized for two reasons. Firstly, for ease of interpretation (given that a multinomial logistic regression without dichotomization yielded similar results), and secondly because the test of parallel lines showed that a logistic regression model was not valid for our data. A hierarchical model structure was adopted in order to provide insights into the relations between the separate variables of interest. First, bivariate relationships were examined using attitudinal factors and subjective health as independent variables. Second, these variables were added simultaneously in the model. Third, socio-demographics were added as control variables. Odds ratios and confidence intervals were inspected and compared. The *p*-value for acceptance and rejection of the null hypotheses in the logistic regressions was set at *p* < 0.05. The Nagelkerke R^2^ and the Cox & Snell R^2^ and goodness-of-fit were assessed using a Likelihood Ratio chi-square test [40, 41].

The data were analysed using STATA 15.0.

## Results

### Sample characteristics

**Fig. 1:**
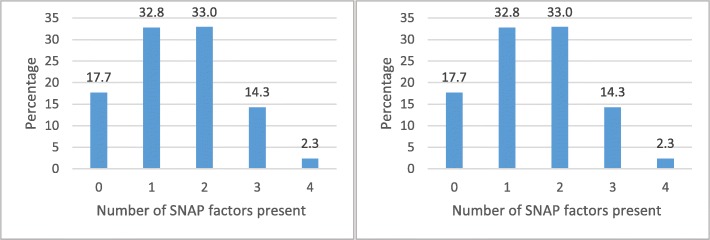
Prevalence of unhealthy SNAP behaviours and cumulative unhealthy SNAP behaviours present

In total, 1006 respondents completed the survey. Respondents who provided inconsistent or impossible values (e.g. SLE lower than current age) were excluded from further analyses. This resulted in a final sample of 989 respondents. Figure 1 shows the prevalence of unhealthy SNAP behaviours and the number of unhealthy SNAP behaviours in this study population (i.e. presence refers to the unhealthy choice). Almost a quarter (23%) of respondents reported smoking, and half of the population did not satisfy the thresholds for a healthy diet or the guidelines of physical activity (respectively 47% and 52%). Almost half of the respondents (49.6%) were engaged in two or more unhealthy SNAP behaviours.

Table 1 shows the socio-demographic characteristics of the study population. Smoking was significantly more concentrated among lower educated people. People with an unhealthy diet were significantly younger. Excessive drinkers were significantly more often women, younger and lower educated than other respondents. Physical inactivity was significantly more common in the youngest age group.

**Table 1:** Sociodemographic factors by unhealthy SNAP behaviours

Characteristics	Total % (N)	Smoking^a^% (N)	Diet^a^% (N)	Alcohol^a^% (N)	Physical inactivity^a^% (N)
Sample	100 (989)	23.0 (227)	46.9 (464)	29.2 (289)	51.7 (511)
**Gender**					
Men	50.7 (501)	24.2 (121)	48.7 (244)	24.6 (123)*	50.5 (253)
Women	49.3 (488)	21.7 (106)	45.1 (220)	34.0 (166)	52.9 (258)
**Age**					
18–34 yrs	15.6 (154)	20.13 (31)	62.3 (96)*	36.4 (56)	66.2 (102)*
35–54 yrs	34.8 (344)	26.2 (90)	53.5 (184)	30.2 (104)	52.0 (179)
55–75 yrs	49.6 (491)	21.6 (106)	37.5 (184)	26.3 (129)	46.8 (230)
**Average age (range)**	51.6 (18–75)	51.4 (18–74)	48.5 (18–73)*	49.9 (18–72)*	50.1 (18–74)*
**Completed education level**					
Low	21.6 (214)	30.4 (65)*	50.5 (108)	38.3 (82)*	52.3 (112)
Middle	48.3 (478)	24.1 (115)	48.5 (232)	28.9 (138)	53.8 (257)
High	30.0 (297)	15.8 (47)	41.8 (124)	23.2 (69)	47.8 (142)

* = significant at *p* < .001 level derived from chi^2^ and ANOVA tests

^a^ Column presents characteristics of respondents engaged in the risky behaviour. Numbers are compared to people who are not engaged in this behaviour

### Combinations of unhealthy SNAP behaviours

All possible combinations of unhealthy SNAP behaviours were present in our sample (Table 2). Men were more frequently engaged in none of the unhealthy SNAP behaviours (19.6% vs. 15.8% in women), but the largest group of men was engaged in two or more unhealthy SNAP behaviours (36.3%), while the largest share of women was engaged in one unhealthy SNAP behaviour (36.1%). People engaged in multiple unhealthy SNAP behaviours were mostly younger. People who reported physical inactivity, either alone or in combination with an unhealthy diet, were significantly higher educated. An unhealthy diet and physical inactivity was the most prevalent combination (16.5%). The frequency of other combinations of unhealthy behaviours was diffuse. Excessive alcohol consumption and smoking share that they are both addictive behaviours and are therefore regularly studied in combination. Hence, for this combination (independently from other presence of other unhealthy SNAP behaviours) we provided further characteristics concerning the attitudinal factors and subjective health in Table 3, however this combination was not included in the regression analysis.

**Table 2:** Cumulative unhealthy SNAP behaviours present and all possible combinations stratified by age, sex and level of education

Cumulative unhealthy SNAP behaviours and combinations			Gender		Age, μ^*^	Completed educational level		
		% (N)	Men	Women		Low	Medium	High
**0**	*No unhealthy SNAP behaviours*	***17.7 (175)***	**19.6 (98)**	**15.8 (77)**	**56.1**	**16.4 (35)**	**15.1 (72)**	**22.9 (68)**
**1**	Smoking (S)	4.2 (42)	4.8 (24)	3.7 (18)	55.2	3.7 (8)	5.0 (24)	3.4 (10)
	Unhealthy diet (N)	7.9 (78)	6.8 (34)	9.0 (44)	49.1	6.0 (13)	8.6 (41)	8.1 (24)
	Excessive drinking (A)	7.4 (73)	5.8 (29)	9.0 (44)	51.3	4.7 (10)	7.7 (37)	8.8 (26)
	Physical inactivity (P)	13.3 (131)	12.2 (61)	14.3 (70)	54.5	8.9 (19)	14.2 (68)	14.8 (44)
	**Total**	**32.8 (324)**	**29.5 (148)**	**36.1 (176)**	**52.6**	**23.4 (50)**	**35.6 (170)**	**35.0 (104)**
**2**	S N	3.9 (39)	5.2 (26)	2.7 (13)	53.1	7.5 (16)	2.9 (14)	3.0 (9)
	S A	1.6 (16)	1.0 (5)	2.3 (11)	57.1	4.2 (9)	1.1 (5)	0.7 (2)
	S P	2.5 (25)	3.0 (15)	2.0 (10)	47.7	1.4 (3)	2.5 (12)	3.4 (10)
	N A	4.2 (41)	4.4 (22)	3.9 (19)	49.3	3.7 (8)	4.2 (20)	4.4 (13)
	N P	16.5 (163)	19.4 (97)	13.5 (66)	48.3	12.6 (27)	17.2 (82)	18.2 (54)
	A P	4.3 (42)	3.4 (17)	5.1 (25)	53.4	7.5 (16)	3.4 (16)	3.4 (10)
	**Total**	**33.0 (326)**	**36.3 (182)**	**29.5 (144)**	**50.4**	**36.9 (79)**	**31.2 (149)**	**33.0 (98)**
**3**	S N A	1.4 (14)	2.0 (10)	0.8 (4)	48.6	1.4 (3)	1.7 (8)	1.0 (3)
	S N P	4.8 (47)	4.6 (23)	4.9 (24)	47.4	5.1 (11)	5.7 (27)	3.0 (9)
	S A P	2.1 (21)	1.6 (8)	2.7 (13)	47.7	2.8 (6)	2.5 (12)	1.0 (3)
	N A P	6.0 (59)	4.4 (22)	7.6 (37)	45.3	9.8 (21)	5.7 (27)	3.7 (11)
	**Total**	**14.3 (141)**	**12.6 (63)**	**16.0 (78)**	**46.7**	**19.2 (41)**	**15.5 (74)**	**8.8 (26)**
**4**	S N A P	**2.3 (23)**	**2.0 (10)**	**2.7 (13)**	**49.7**	**4.2 (9)**	**2.7 (13)**	**0.3 (1)**
	**Total**	**100 (989)**	**100 (501)**	**100 (488)**	**51.6**	**100 (214)**	**100 (478)**	**100 (297)**

^*^ = Significant at *p* < .001 level derived from an ANOVA test

**Table 3:** Group mean values of attitudinal factors and subjective health by unhealthy SNAP behaviours, tested for significance

Prevalence behaviour		% (N)	Present orientation (CFC–I)	Future orientation (CFC–F)	Risk attitude (HRAS-6)	SRH	SLE
S	Risky	23.0 (227)	4.0^*^	4.1^*^	20.5^*^	6.6^*^	80.4^*^
	Healthy	77.0 (762)	3.7	4.4	17.0	7.1	84.1
N	Risky	46.9 (464)	3.8	4.3	19.5^*^	6.7^*^	82.2^*^
	Healthy	53.1 (525)	3.7	4.4	16.2	7.2	84.2
A	Risky	29.2 (289)	3.9	4.4	18.0	6.7^*^	81.9
	Healthy	70.8 (700)	3.7	4.4	17.7	7.1	83.8
P	Risky	51.7 (511)	3.8	4.4	18.8_*_	6.7_*_	82.6
	Healthy	48.3 (478)	3.7	4.4	16.7	7.3	84.0
**Lifestyle index**							
0		17.7 (175)	3.6^*^	4.6_*_	14.7_*_	7.6_*_	85.5^*^
1		32.8 (324)	3.7	4.4	16.9	7.2	84.2
2		33.0 (326)	3.8	4.3	18.6	6.8	83.1
3		14.3 (141)	4.1	4.2	21.1	6.4	80.4
4		2.3 (23)	4.2	4.5	22.1	5.5	73.7
**Prevalent combinations of unhealthy behaviours**							
P N		16.5 (163)	3.6	4.4	19.2^*^	6.8	82.9
S A ^a^		7.5 (74)	4.1	4.1	22.1^*^	6.0^*^	77.1^*^
**Total**		**100 (989)**	**3.7**	**4.4**	**17.8**	**7.0**	**83.3**

^*^ = Significant at *p* < .001 level derived from an ANOVA test

^a^ Combination independent from engagement in other unhealthy SNAP behaviours

### Unhealthy SNAP behaviours in relation to attitudinal factors and subjective health

Table 3 provides average scores of the attitudinal factors and subjective health. Smokers had the highest score on the CFC-I, indicating a high focus on immediate consequences. Smokers also had the highest score on the HRAS-6: they were more risk seeking in the health domain than non-smokers. SRH and SLE were significantly lower for people engaged in an unhealthy SNAP behaviour. Smokers reported the lowest subjective health values.

The presence of multiple unhealthy SNAP behaviours was associated with a higher CFC-I score. The absence of an unhealthy SNAP behaviour was associated with a higher CFC-F score. SRH and SLE decreased significantly when the number of unhealthy SNAP behaviours increased. A noteworthy finding is the gap of 2.1 points in SRH (scale 0–10) between people with zero and four unhealthy SNAP behaviours. Likewise, the discrepancy in SLE between zero and four unhealthy SNAP behaviours was remarkable at almost 12 years (86 versus 74).

The most prevalent combination (unhealthy diet and physical inactivity) showed values comparable to the study population averages on all characteristics. The combination smoking and excessive alcohol consumption (SA, *n* = 16; SNA, *n* = 14; SAP, *n* = 21; SNAP, *n* = 23) occurred in only 7.5% of the sample. This group is significantly more focussed on immediate consequences and less on future consequences. They also appear to be relatively more risk seeking and had relatively low values on both SRH and SLE.

### Logistic regressions

Odds ratios of the bivariate analysis, crude analysis and adjusted associations of the individual and clustered unhealthy SNAP behaviours with the attitudinal factors and subjective health are presented in the Appendix (as models M1, M2 and M3, respectively). Table 4 summarizes these results and presents the adjusted associations (which coincide with the models M3 in the Appendix).

**Table 4:** Odds ratio’s with 95% confidence intervals for the presence of unhealthy SNAP behaviours (*N* = 984)

Variable	SNAP behaviours^a^								Combinations of unhealthy behaviours^a^			
	Smoking		Unhealthy diet		Excessive drinking		Physical inactivity		Physical inactivity & unhealthy diet		Lifestyle index^b^	
	OR (p)	CI	OR (p)	CI	OR (p)	CI	OR (p)	CI	OR (p)	CI	OR (p)	CI
CFC-I	1.30* (.00)	1.08–1.56	1.11 (.18)	0.95–1.30	1.18* (.04)	1.00–1.38	1.05 (.51)	0.91–1.22	0.81* (.04)	0.66–0.99	1.19* (.03)	1.01–1.39
CFC-F	0.95 (.63)	0.77–1.18	1.04 (.66)	0.87–1.25	1.09 (.37)	0.90–1.31	1.20* (.03)	1.01–1.44	0.92 (.50)	0.72–1.17	1.05 (.60)	0.87–1.27
HRAS – 6	1.12* (.00)	1.08–1.15	1.09* (.00)	1.06–1.12	0.99 (.35)	0.96–1.01	1.06* (.00)	1.03–1.09	1.04* (.02)	1.00–1.09	1.09* (.00)	1.06–1.12
Self-rated health	1.00 (.95)	0.89–1.12	0.89* (.00)	0.81–0.98	0.85* (.00)	0.77–0.94	0.84* (.00)	0.76–0.92	0.92 (.20)	0.82–1.04	0.81* (.00)	0.74–0.90
Subjective life expectancy	0.97*(.00)	0.95–0.99	1.00 (.53)	0.98–1.01	0.99 (.15)	0.97–1.00	1.00 (.98)	0.98–1.01	1.00 (.65)	0.99–1.02	0.99 (.18)	0.97–1.00
Age	1.01 (.38)	0.99–1.02	0.97* (.00)	0.86–0.98	0.98* (.00)	0.97–0.99	0.99* (.04)	0.98–0.99	0.98* (.00)	0.97–0.99	0.98* (.00)	0.97–0.99
Sex												
Male	1.00		1.00		1.00		1.00		1.00		1.00	
Female	0.86 (.36)	0.62–1.19	0.75* (.00)	0.57–0.99	1.45* (.01)	1.09–1.90	1.06 (.67)	0.81–1.38	0.58* (.00)	0.41–0.83	0.77 (.07)	0.59–1.02
Education level												
Low	1.00		1.00		1.00		1.00		1.00		1.00	
Middle	0.74 (.14)	0.50–1.10	0.75 (.11)	0.52–1.06	0.67* (.03)	0.47–0.97	0.99 (.95)	0.70–1.40	1.18 (.52)	0.71–1.93	0.53* (.00)	0.37–0.77
High	0.49* (.00)	0.30–0.80	0.58* (.00)	0.39–0.88	0.55* (.00)	0.36–0.85	0.79 (.24)	0.53–1.17	1.22 (.48)	0.70–2.11	0.42* (.00)	0.28–0.64
Nagelkerke R^2^	0.166		0.163		0.070		0.080		0.058		0.196	
Cox and Snell R^2^	0.110		0.122		0.049		0.060		0.034		0.147	

* = Significant at *p* < .05 level

^a^ Absent (0) versus present (1)

^b^ 0 or 1 unhealthy SNAP behaviour present versus 2, 3 or 4 unhealthy SNAP behaviours present

#### Attitudinal factors

Smoking, excessive drinking, and the lifestyle index were significantly associated with CFC-I scores, which increased the odds by 30% (95% CI; 1.08–1.56), 18% (95% CI; 1.00–1.38) and 19% (95% CI; 1.01–1.39) respectively. The combination of being physically inactive and having an unhealthy diet was significantly associated with CFC-I scores, which decreased the odds by 19% (95% CI; 0.66–0.99). Only physical inactivity was significantly associated with CFC-F scores, which increased the odds by 20% (95% CI; 1.01–1.44). Smoking, an unhealthy diet, and physical inactivity were significantly associated with HRAS-6 scores, which increased the odds by 12% (95% CI; 1.08–1.15), 9% (95% CI; 1.06–1.12) and 6% (95% CI; 1.03–1.09), respectively. The combination of physical inactivity and an unhealthy diet, and the lifestyle index were also significantly associated with HRAS-6 scores, which increased the odds by 4% (95% CI; 1.00–1.09) and 9% (95% CI; 1.06–1.12), respectively.

#### Subjective health

An unhealthy diet, excessive drinking, and physical inactivity were significantly associated with SRH, which decreased the odds by 11% (95% CI; 0.81–0.98), 15% (95% CI; 0.77–0.94); and 16% (95% CI; 0.76–0.92) respectively. The lifestyle index was also significantly associated with SRH, which decreased the odds by 19% (95% CI; 0.74–0.90). Only smoking was significantly associated with SLE, which decreased the odds by 3% (95% CI; 0.95–0.99).

#### Sociodemographic characteristics

The socio-demographic control variables had the following associations. All SNAP behaviours except smoking, the combination of physical inactivity and an unhealthy diet, and the lifestyle index were significantly associated with age, which decreased the odds by between 1% and 3%. An unhealthy diet and the combination of physical inactivity and an unhealthy diet were significantly associated with being a female, which decreased the odds by 25% (0.57–0.99) and 42% (95% CI; 0.41–0.83), respectively. Excessive drinking was also significantly associated with being a female, which increased the odds by 45% (95% CI; 1.09–1.90). Finally, smoking and an unhealthy diet were significantly associated with high level of education compared to low level of education, which decreased the odds by 51% (95% CI; 0.30–0.80) and 42% (95% CI; 0.39–0.88), respectively. Excessive drinking and the lifestyle index were significantly associated with both medium and high level of education compared to low level of education, which decreased the odds by 33% (95% CI; 0.47–0.97) and 45% (95% CI; 0.36–0.85) for excessive drinking, and decreased the odds by 47% (95% CI; 0.37–0.77) and 58% (95% CI; 0.28–0.64) for the lifestyle index.

## Discussion

In the current study, unhealthy SNAP behaviours were studied independently and in combination with each other. The prevalence of smoking, unhealthy diet and physical inactivity was comparable to figures for the general Dutch population [42]. However, the prevalence of excessive alcohol consumption (29%) was considerably higher than reported in official Dutch population statistics (9%) [42]. Half of our study population was engaged in two or more unhealthy SNAP behaviours. The most prevalent combination was an unhealthy diet combined with physical inactivity (17%). Smoking, drinking excessively and the lifestyle index were significantly associated with an increased focus on the immediate consequences of behaviour (i.e., the CFC-I). On the other hand, we also found that being physical inactive was significantly associated with an increased focus on the future consequences of behaviour (i.e., the CFC-F). This latter finding is contradictory to what one may expect. These findings may have implications for public health policy, but need to be confirmed longitudinally.

Applying the two-factor structure of the CFC in our regression analysis revealed that smokers were significantly more oriented on immediate consequences compared to non-smokers. However, we did not find a future-oriented attitude among non-smokers. This finding underlines the added value of a two factor structure for the CFC. Previous studies also found that smokers are more present oriented, both when using the CFC as one scale [43] or two sub-scales [17]. The CFC also has been used in relation to healthy eating, physical activity and BMI [17, 34, 43, 44]. Our results confirm previous findings, indicating that people engaged in unhealthy behaviour(s) are especially oriented towards the immediate consequences of their behaviour. This finding does not apply to physical activity, however. We even found a more future oriented attitude for physically inactive people. This finding is counter intuitive since physical activity typically provides gains on the long term. Doing sports is also found to bring positivity and reward just after the exercise and therefore a more present-oriented attitude may also suit athletic people [45]. In this study the question concerning physical activity not only involved “physical exercise” or “sports” but also walking or climbing stairs. Therefore, an appropriate interpretation of this finding is complicated. We found that the people who had both an unhealthy diet and were physical inactive were significantly less oriented on immediate consequences. This implies that time orientation for unhealthy SNAP behaviours can differ between a single behaviour and a particular combination of behaviours. Findings regarding risk attitude were in line with the general risk attitude hypothesis. People engaged in an unhealthy SNAP behaviour, except for excessive drinking, were more risk seeking than those people not engaged in this unhealthy SNAP behaviour. This association was persistent when considering multiple unhealthy SNAP behaviours. The HRAS-6 (the instrument we applied for risk attitude assessment) has recently been introduced and was shown to be a valid and reliable measure of health-risks attitudes [38]. The different results found for alcohol consumption could be related to the high percentage of excessive alcohol drinkers in our population, which might be less representative of problematic drinking populations.

The presence of unhealthy SNAP behaviours was associated with significantly lower SRH, although for smoking this was not confirmed in the regression analyses. Two potential explanations can be put forward. First, in the regression analyses we controlled for the potential differences in SRH attributed to co-variates. It is conceivable that the co-variates (sociodemographic-characteristics) explain differences in SRH more than smoking does. Second, it is suggested in the literature that smokers tend to understimate short-term risks of smoking [46]. This phenomenon might be reflected in the current SRH status of smokers. The clustering of unhealthy SNAP behaviours and the association of these clusters to SRH has been studied before [47]. Conry et al., (2011) found that respondents with multiple unhealthy SNAP behaviours reported less good SRH scores than respondents with less unhealthy SNAP behaviours.

In the logistic regression analyses, the association between unhealthy SNAP behaviours and SLE only remained significant for smoking. This may imply that people are aware of negative long term health consequences of smoking, which has also been found in previous studies [48–51]. Hence, reiterating long-term consequences in preventive messages may have little effect on behaviour, which is emphasized by the finding that people engaged in an unhealthy SNAP behaviour are more focussed on immediate rather than future consequences. Two other remarks can be made about the association between SLE and unhealthy behaviour. First, a person engaged in risky behaviour might already experience decreased health due to the chosen lifestyle, which in turn negatively affects SLE. Second, one might expect a lower life expectancy when family members on average died relatively young [27, 52]. Unhealthy habits may then be expected to not or only marginally affect the already low life expectancy.

### Study limitations and strengths

A number of limitations of this study need to be mentioned. First, the unhealthy SNAP behaviours were operationalised through dichotomisation, with people either having the risk factor or not. Cut-off points from national guidelines were used to do so. It is important to note that these cut-off points remain somewhat arbitrary and our findings may be sensitive to the cut-off point chosen. For instance, using the national guidelines we observed a considerably higher prevalence of excessive drinking in our sample as compared to national statistics. However, this prevalence would have been even higher if we had adopted alternative, often stricter, international guidelines for excessive drinking, for example the classification defined by the National Institute on Alcohol Abuse and Alcoholism (NIAAA). Second, unhealthy SNAP behaviours were self-reported, which might result in an under- or over estimation of certain habits. Third, alcohol consumption in this study population substantially differed from that of the Dutch population (29% versus 9%). This may highlight that the panel of the sampling agency reached a particular selection of Dutch individuals. This limits the generalisability of our findings. Fourth, we do not know how many people declined to participate in the survey, or dropped-out, as this information was not made available by the survey company for commercial reasons. This information is important to examine potential selection bias in the sample, and given its unavailability, we cannot rule out potential selection bias. Fifth, due to the cross-sectional design of our study, we could not investigate causal relationships. For instance, our data does not allow us to investigate whether people become more present-oriented because they smoke, or whether people become smokers more easily because they are more present-oriented. While examining this further is important, knowing the associations may already be useful for designing interventions and future research.

Several strengths of this study also deserve to be highlighted. Except for the over-representation of excessive alcohol drinkers, our sample appears to be fairly representative of the adult population of the Netherlands in terms of sex, age, educational level and unhealthy SNAP behaviours. The study sample was large with almost 1000 respondents. Moreover, our dataset was relatively rich in terms of the wide variety of included variables. Finally, we tested different clustering techniques to identify combinations of unhealthy SNAP behaviours in the sample, but in the end opted for the simpler and more straightforward approach presented here. The results for this approach were essentially the same and had a somewhat clearer interpretation. In addition, this approach for clustering the unhealthy SNAP behaviours is easier to communicate to a general audience with a less advanced background in statistics.

## Conclusions

Our findings emphasize the relevance of taking a holistic approach to health prevention rather than focusing on a single behaviour only. We conclude from our study that people who were engaged in none or one unhealthy SNAP behviour differ significantly on attitudunal factors and subjective health from people engaged in multiple unhealthy SNAP behaviours. However, the specific combination of unhealthy SNAP behaviours also seems to matter, as the most prevalent combination (physical inactivity and an unhealthy diet) showed an opposite relationship with time orientation as compared to the lifestyle index. People who engage in just one unhealthy SNAP behaviour may lack willingness to change because they feel they compensate for this behaviour with other healthy habits. On the other hand, people engaged in multiple unhealthy SNAP behaviours might be less easily affected by health promotion messages. Policy or specific interventions targeting lifestyle could incorporate the attitudinal factors analysed in this study to increase the probability of reaching the desired target group.

## Data Availability

The dataset used for the current study are available from the corresponding author on reasonable request.

## Appendix

### Bivariate, crude and adjusted associations with unhealthy SNAP behaviours

**Table 5:** Odds ratio's with 95% confidence intervals for smoking

	Smoking					
	M1		M2		M3	
	OR (p)	CI	OR (p)	CI	OR (p)	CI
CFC	0.53* (.00)	0.43–0.66				
CFC-I	1.50* (.00)	1.28–1.76	1.37* (.00)	1.14–1.64	1.30* (.00)	1.08–1.56
CFC-F	0.66 * (.00)	0.55–0.79	0.90 (.33)	0.73–1.11	0.95 (.63)	0.77–1.18
HRAS-6	1.13* (.00)	1.09–1.16	1.10* (.00)	1.07–1.14	1.12* (.00)	1.08–1.15
Self-rated health	0.83* (.00)	0.76–0.91	0.97 (.53)	0.86–1.08	1.00 (.95)	0.89–1.12
Subjective life expectancy	0.96* (.00)	0.94–0.97	0.97* (.00)	0.95–0.99	0.97* (.00)	0.95–0.99
Age					1.00 (.38)	0.99–1.02
Sex						
Male					1.00	
Female					0.86 (.36)	0.62–1.19
Education level						
Low					1.00	
Middle					0.74 (.14)	0.50–1.10
High					0.49* (.00)	0.30–0.80
Nagelkerke R^2^			0.151		0.166	

M1: Bivariate associations of dependent variable with single characteristics

M2: Characteristics corrected for each other

M3: Odds ratios adjusted for socio demographic characteristics of respondents

^*^ = Significant at *p* < 0.05 level

**Table 6:** Odds ratio's with 95% confidence intervals for an unhealthy diet

	Unhealthy diet					
	M1		M2		M3	
	OR (p)	CI	OR (p)	CI	OR (p)	CI
CFC	0.76* (.00)	0.64–0.91				
CFC-I	1.19* (.00)	1.04–1.36	1.15 (.07)	0.99–1.33	1.11 (.18)	0.95–1.30
CFC-F	0.85* (.02)	0.72–0.97	1.09 (.35)	0.91–1.30	1.04 (.66)	0.87–1.25
HRAS-6	1.12* (.00)	1.09–1.15	1.11* (.00)	1.08–1.14	1.09* (.00)	1.06–1.12
Self-rated health	0.82* (.00)	0.75–0.89	0.90* (.02)	0.82–0.99	0.89* (.00)	0.81–0.98
Subjective life expectancy	0.98* (.00)	0.96–0.99	0.99 (.36)	0.98–1.00	1.00 (.53)	0.98–1.01
Age					0.97* (.00)	0.86–0.98
Sex						
Male					1.00	
Female					0.75* (.00)	0.57–0.99
Education level						
Low					1.00	
Middle					0.75 (.11)	0.52–1.06
High					0.58* (.00)	0.39–0.88
Nagelkerke R^2^			0.124		0.163	

M1: bivariate associations of dependent variable with single characteristics.

M2: multivariate associations of dependent variable with characteristics.

M3: odds ratios adjusted for socio demographic characteristics of respondents.

* = significant at *p* < .05 level

**Table 7:** Odds ratio's with 95% confidence intervals for excessive drinking

	Excessive drinking					
	M1		M2		M3	
	OR(p)	CI	OR(p)	CI	OR(p)	CI
CFC	0.84 (.08)	0.70–1.02				
CFC-I	1.18* (.02)	1.02–1.37	1.21* (.01)	1.04–1.41	1.18* (.04)	1.00–1.38
CFC-F	0.96 (.64)	0.82–1.13	1.06 (.53)	0.88–1.27	1.09 (.37)	0.90–1.31
HRAS-6	1.01 (.39)	0.99–1.03	0.99 (.44)	0.96–1.02	0.99 (.35)	0.96–1.01
Self-rated health	0.83* (.00)	0.76–0.90	0.84* (.00)	0.76–0.92	0.85* (.00)	0.77–0.94
Subjective life expectancy	0.98* (.00)	0.96–0.99	0.99 (.10)	0.97–1.00	0.99 (.15)	0.97–1.00
Age					0.98* (.00)	0.97–0.99
Sex						
Male					1.00	
Female					1.45* (.01)	1.09–1.90
Education level						
Low					1.00	
Middle					0.67* (.03)	0.47–0.97
High					0.55* (.00)	0.36–0.85
Nagelkerke R^2^			0.039		0.070	

M1: bivariate associations of dependent variable with single characteristics

M2: multivariate associations of dependent variable with characteristics

M3: odds ratios adjusted for socio demographic characteristics of respondents

*** = significant at *p* < .001 level; ** = significant at *p* < 0.005 level; * = significant at *p* < .05 level

**Table 8:** Odds ratio's with 95% confidence intervals for physical inactivity

	Physical inactivity					
	M1		M2		M3	
	OR(p)	CI	OR(p)	CI	OR(p)	CI
CFC	0.93 (.41)	0.78–1.10				
CFC-I	1.08 (.25)	0.95–1.23	1.07 (.39)	0.92–1.23	1.05 (.51)	0.91–1.22
CFC-F	0.99 (.91)	0.85–1.15	1.20* (.03)	1.01–1.43	1.20* (.03)	1.01–1.44
HRAS-6	1.07* (.00)	1.04–1.09	1.01* (.00)	1.03–1.09	1.06* (.00)	1.03–1.09
Self-rated health	0.80* (.00)	0.73–0.87	0.84* (.00)	0.76–0.92	0.84* (.00)	0.76–0.92
Subjective life expectancy	0.98* (.01)	0.97–1.00	1.00 (.84)	0.98–1.01	1.00 (.98)	0.98–1.01
Age					0.99* (.04)	0.98–0.99
Sex						
Male					1.00	
Female					1.06 (.67)	0.81–1.38
Education level						
Low					1.00	
Middle					0.99 (.95)	0.70–1.40
High					0.79 (.24)	0.53–1.17
Nagelkerke R^2^			0.071		0.080	

M1: bivariate associations of dependent variable with single characteristics

M2: multivariate associations of dependent variable with characteristics

M3: odds ratios adjusted for socio demographic characteristics of respondents

* = significant at *p* < .05 level

**Table 9:** Odds ratio's with 95% confidence intervals for physical inactivity and smoking

	Physical inactivity & unhealthy diet					
	M1		M2		M3	
	OR (p)	CI	OR (p)	CI	OR (p)	CI
CFC	1.10 (.42)	0.87–1.38				
CFC-I	0.87 (.11)	0.72–1.03	0.80* (.03)	0.66–0.98	0.81* (.04)	0.66–0.99
CFC-F	0.96 (.68)	0.78–1.17	1.00 (.96)	0.80–1.27	0.92 (.50)	0.72–1.17
HRAS-6	1.06* (.00)	1.02–1.09	1.06* (.00)	1.02–1.10	1.04* (.02)	1.00–1.09
Self-rated health	0.91 (.09)	0.82–1.01	0.95 (.42)	0.85–1.07	0.92 (.20)	0.82–1.04
Subjective life expectancy	1.00 (.60)	0.98–1.01	1.00 (.66)	0.99–1.02	1.00 (.65)	0.99–1.02
Age					0.98* (.00)	0.97–0.99
Sex						
Male					1.00	
Female					0.58* (.00)	0.41–0.83
Education level						
Low					1.00	
Middle					1.18 (.52)	0.71–1.93
High					1.22 (.48)	0.70–2.11
Nagelkerke R^2^			0.032		0.058	

M1: bivariate associations of dependent variable with single characteristics

M2: multivariate associations of dependent variable with characteristics

M3: odds ratios adjusted for socio demographic characteristics of respondents

* = significant at *p* < .05 level

**Table 10:** Odds ratio's with 95% confidence intervals for the lifestyle index

	Lifestyle index^**a**^					
	M1		M2		M3	
	OR (p)	CI	OR (p)	CI	OR (p)	CI
CFC	0.68* (.00)	0.57–0.81				
CFC-I	1.31* (.00)	1.14–1.49	1.25* (.00)	1.07–1.46	1.19* (.03)	1.01–1.39
CFC-F	0.79* (.00)	0.68–0.92	1.07 (.48)	0.89–1.27	1.05 (.60)	0.87–1.27
HRAS-6	1.12* (.00)	1.09–1.14	1.10* (.00)	1.07–1.13	1.09* (.00)	1.06–1.12
Self-rated health	0.73* (.00)	0.67–0.80	0.81* (.00)	0.73–0.89	0.81* (.00)	0.74–0.90
Subjective life expectancy	0.97* (.00)	0.96–0.98	0.99 (.13)	0.97–1.00	0.99 (.18)	0.97–1.00
Age					0.98* (.00)	0.97–0.99
Sex						
Male					1.00	
Female					0.77 (.07)	0.59–1.02
Education level						
Low					1.00	
Middle					0.53* (.00)	0.37–0.77
High					0.42* (.00)	0.28–0.64
Nagelkerke R^2^			0.157		0.196	

M1: bivariate associations of dependent variable with single characteristics

M2: multivariate associations of dependent variable with characteristics

M3: odds ratios adjusted for socio demographic characteristics of respondents

* = significant at *p* < .05 level

^a^: 0 or 1 unhealthy SNAP behaviour present versus 2, 3 or 4 unhealthy SNAP behaviours present

## Abbreviations

CFC – F: Consideration of Future Consequences Future
CFC – I: Consideration of Future Consequences Immediate
CFC: Consideration of Future Consequences
HRAS: Health Risk Attitude Scale
NCDs: Non Communicable Diseases
SLE: Subjective life expectancy
SNAP: Smoking, Nutrition, Alcohol consumption, Physical activity
SRH: Self Rated Health
VAS: Visual Analogue Scale
