# Correction to: How do combinations of unhealthy behaviors relate to attitudinal factors and subjective health among the adult population in the Netherlands?

**DOI:** 10.1186/s12889-020-09935-4

**Published:** 2020-11-27

**Authors:** Charlotte M. Dieteren, Werner B. F. Brouwer, Job van Exel

**Affiliations:** 1 Erasmus University Rotterdam, Erasmus School of Health Policy & Management, P.O. Box 1738, 3000 DR Rotterdam, the Netherlands; 2 Erasmus University Rotterdam, Erasmus School of Economics, Rotterdam, The Netherlands

**Correction to: BMC Public Health 20, 441 (2020)**

**https://doi.org/10.1186/s12889-020-8429-y**

It was highlighted that in the original article [1] the graphs in Fig. 1 were duplicated. This Correction article shows the correct Fig. 1. The original article has been updated.

**Fig. 1:**
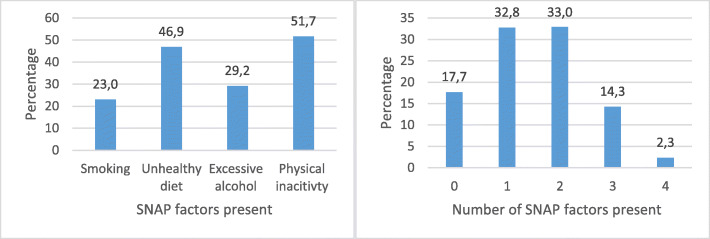
Prevalence of SNAP factors and cumulative SNAP factors present
